# Gross Cystic Disease Fluid Protein 15 in Stratum Corneum Is a Potential Marker of Decreased Eccrine Sweating for Atopic Dermatitis

**DOI:** 10.1371/journal.pone.0125082

**Published:** 2015-04-28

**Authors:** Koji Kamiya, Jun-Ichi Sakabe, Hayato Yamaguchi, Takahiro Suzuki, Tsuyoshi Yatagai, Masahiro Aoshima, Taisuke Ito, Yoshiki Tokura

**Affiliations:** Department of Dermatology, Hamamatsu University School of Medicine, Hamamatsu, Japan; CNRS-University of Toulouse, FRANCE

## Abstract

It is well known that eccrine sweating is attenuated in patients with atopic dermatitis (AD). We have reported by using proteome analysis that gross cystic disease fluid protein 15 (GCDFP15), a substance secreted from eccrine sweat glands, is decreased in tape-stripped stratum corneum (SC) samples from AD patients. The aim of this study was to evaluate GCDFP15 production by eccrine glands with SC samples and to assess sweating in AD. SC samples were obtained from 51 healthy control (HC) and 51 AD individuals. Sweat samples were from 18 HC and 12 AD subjects. GCDFP15 was quantified by ELISA. By immunohistochemistry, the expression of GCDFP15 in eccrine glands was examined in normal and AD skin specimens. To identify GCDFP15-producing cells, double immunofluorescence staining for GCDFP15 and S100 protein was performed in frozen sections. To address the mechanism underlying the decreased eccrine sweating in AD patients, we examined the expression of cholinergic receptor M3 (CHRM3), a receptor for acetylcholine-induced sweating, in eccrine sweat glands. The amounts of GCDFP15 in the SC extracts were significantly lower in AD than HC (*P* < 0.0001). The sweat samples from AD patients also had lower levels of GCDFP15 concentration (*P* < 0.05). Immunohistochemistry showed positive GCDFP15 staining in the eccrine gland secretory cells and the ductal and acrosyringial lumen in normal skin, but AD lacked clear staining. Immunofluorescence staining revealed that GCDFP15 was co-expressed with S100 protein, suggesting that the clear cell of eccrine glands produces GCDFP15. Finally, we found that the expression of CHRM3 was depressed in AD, suggesting contribution to the low sweating. The SC of AD patients contains a low amount of GCDFP15 due to both low sweating and low GCDFP15 concentration in the sweat. GCDFP15 in SC is a potential marker for dysregulated sweating in AD.

## Introduction

Sweating plays a significant role in human skin homeostasis with moisturizing effects [1], antimicrobial effects [2], and thermoregulatory function [3]. Atopic dermatitis (AD) is a chronic inflammatory skin disease, characterized by eczematous skin lesions and intense pruritus [4]. Both skin barrier and immunological abnormalities are involved in the pathogenesis of AD, and impairment of stratum corneum (SC) barrier, as represented by filaggrin deficiency, induces allergic responses to external antigens [5,6]. It has been known that sweating is dysregulated and reduced in AD [7,8]. AD patients, especially those with the IgE-high extrinsic type, have low levels of skin surface hydration [9]. The hypo-hydration is caused mainly by decreased water-holding capacity due to the impaired barrier, but the reduced sweating also may contribute to the perturbed surface hydration. In fact, it has been reported that acetylcholine-mediated sweating is impaired in AD patients [10,11].

Gross cystic disease fluid protein 15 (GCDFP15), also known as prolactin-inducible protein, is expressed in normal exocrine organs, such as sweat, salivary and lacrimal glands [12,13]. GCDFP15 is also expressed in benign and malignant human breast tumors. It is highly specific for mammary differentiation and the expression is regulated by the androgen receptor [14]. Consistently, GCDFP15 is highly expressed in androgen receptor-positive tumors [15]. More clinically, GCDFP15 is frequently used as an immunohistochemical marker for evaluation of a potential mammary origin of metastatic carcinoma, and it is a positive prognostic factor in patients with breast cancer [15]. In addition, it has been demonstrated that GCDFP15 is a novel biomarker in other adenomatous diseases [16,17,18].

Recently, our proteome study disclosed wide-ranging proteins in SC extracts, and AD was shown to be a representative target for this analysis [19]. By using this mass spectrometric analysis, we found that the amounts of GCDFP15 were decreased in SC samples from AD patients. Given that GCDFP15 is produced by eccrine sweat glands, the reduction of GCDFP15 might be attributable to attenuated eccrine sweating. In Sjögren syndrome, the low expression of GCDFP15 reflects both the anatomical damage and the functional impairment of salivary glands [18], and AD possibly shares the sweating disorder with Sjögren syndrome [20].

In this study, we aimed to evaluate GCDFP15 production with SC samples and to assess sweating in AD. GCDFP15 was quantified in SC and sweat samples from AD and healthy individuals, and its expression was examined in AD and normal skin specimens. Results suggest that GCDFP15 is a potential marker of disordered eccrine sweating for AD.

## Materials and Methods

### Collection of SC samples

The study was in accordance with the Declaration of Helsinki. The study was approved by the Ethical Committee of Hamamatsu University School of Medicine and performed at the University Hospital of Hamamatsu University School of Medicine. Written informed consent was obtained from all subjects. Human SC samples were obtained from 51 normal volunteers (Table 1, 29 men and 22 women; mean ± SD age, 32.0 ± 7.4 years, 29 men; mean ± SD age, 31.0 ± 7.6 years, 22 women; mean ± SD age, 33.3 ± 6.9 years) who had no past medical histories and 51 AD cases (Table 1, 31 men and 20 women; mean ± SD age, 32.4 ± 8.9 years, 31 men; mean ± SD age, 32.8 ± 8.9 years, 20 women; mean ± SD age, 31.8 ± 8.7 years). The diagnosis of AD was made by qualified dermatologists at the University Hospital of Hamamatsu University School of Medicine, according to the international consensus criteria [21]. AD patients were treated by topical corticosteroids with or without antihistamines. In order to eliminate the effect of topical application, tape stripping was performed at least 24 h after the last topical application and at least 12 h after washing with soap. On sampling, the skin with severe eczema, moderate to strong lichenification, excoriation, crust, and secondary infection and the skin in joint areas were avoided. The SC sample was obtained by stripping, using the Nichiban cellophane tape (organic solvent-stable tape with organic solvent-soluble adhesive; Nichiban, Tokyo, Japan), as reported previously [19]. In this non-invasive procedure, the tape was applied to the flexor surface of the subjects' forearm and upper arm. SC samples were obtained from 12 different sites per individual using 10 cm length of the tape and were immediately stored at -20°C until treatment with toluene. When the tape was dipped in 10 ml of toluene, all adhesives were dissolved and any attached SC was suspended. After the insoluble tape backing was removed, the sample was centrifuged at 3000 rpm for 15 min. The precipitate was washed with 5 ml of toluene 6 times to remove any residual adhesive. After the toluene treatment, the purified samples were air dried, weighed, and kept at -20°C. Dried SC samples were dissolved by 1% SDS lysis buffer (50 mM Tris HCl, pH 6.8, and 1% SDS) in 1.5 ml protein LoBind tube (Eppendorf, Hamburg, Germany). The samples were then homogenated and sonicated for 20 min (interval, 30 s; sonication, 30 s). The suspension was centrifuged at 13,000 rpm for 15 min at 4°C. The supernatant was collected.

**Table 1:** Collection of stratum corneum samples and sweat samples from healthy controls and atopic dermatitis cases.

Stratum corneum				Sweat			
Age (years, mean ± SD)		Age (years, mean ± SD)		Age (years, mean ± SD)		Age (years, mean ± SD)	
HC (N = 51)	32.0 ± 7.4	AD (N = 51)	32.4 ± 8.9	HC (N = 18)	21.0 ± 2.4	AD (N = 12)	36.8 ± 10.8
Male (N = 29)	31.0 ± 7.6	Male (N = 31)	32.8 ± 8.9	Male (N = 4)	23.8 ± 3.6	Male (N = 8)	36.6 ± 11.5
Female (N = 22)	33.3 ± 6.9	Female (N = 20)	31.8 ± 8.7	Female (N = 14)	20.3 ± 0.9	Female (N = 4)	37.3 ± 7.3

HC: healthy control.

AD: atopic dermatitis.

### Collection of Sweat samples

This study was in accordance with the Declaration of Helsinki. The sampling was approved by the Ethical Committee of Hamamatsu University School of Medicine, and written informed consent was obtained from all subjects. Sweat samples were obtained from the forearms and upper arms of 18 normal volunteers (Table 1, 4 men and 14 women; mean ± SD age, 21.0 ± 2.4 years, 4 men; mean ± SD age, 23.8 ± 3.6 years, 14 women; mean ± SD age, 20.3 ± 0.9 years) who had no past medical histories and 12 AD cases (Table 1, 8 men and 4 women; mean ± SD age, 36.8 ± 10.8 years, 8 men; mean ± SD age, 36.6 ± 11.5 years, 4 women; mean ± SD age, 37.3 ± 7.3 years). The forearms and upper arms were washed clean and wrapped with a plastic bag for 10–15 min. A total of 1 ml of sweat sample was collected after exercise load and heat load. When the test subjects felt physically stressful during the process, the exercise load was discontinued. The collected sweat was filtered with 0.45 μm diameter membrane (Millex, Merck Millipore, Billerica, MA).

### GCDFP15 ELISA

The expression levels of GCDFP15 were evaluated using Human Prolactin Inducible Protein ELISA Kit (Cusabio; Wuhan, China) [22]. According to the assay procedure, 100 μl each of dilutions of standard (40 ng/ml, 20 ng/ml, 10 ng/ml, 5 ng/ml, 2.5 ng/ml, 1.25 ng/ml, 0.625 ng/ml), blank (Standard Diluent, 0 ng/ml), SC samples and sweat samples was added to the wells. After 2 h incubation at 37°C, the liquid of each well was removed and 100 μl of Detection Reagent A working solution was added to each well. After 1 h incubation at 37°C, each well was washed with Wash Solution and 100 μl of Detection Reagent B working solution was added. After 30 min incubation at 37°C, each well was washed with Wash Solution and 90 μl of Substrate Solution was added. The wells were incubated for 25 min at 37°C. After adding 50 μl of Stop Solution to each well, the optical density at 450 nm (OD_450_) was measured immediately. The concentration of GCDFP15 was determined by comparing the OD_450_ of the samples to the calibration standard curve.

### Immunohistochemical staining

This study was in accordance with the Declaration of Helsinki. The sampling was approved by the Ethical Committee of Hamamatsu University School of Medicine, and written informed consent was obtained from all subjects. Skin samples were obtained by qualified dermatologists using a disposable sterile 3-mm punch biopsy (Maruho, Osaka, Japan) at the University Hospital of Hamamatsu University School of Medicine. AD skin samples were obtained from eczematous lesions in the trunk and flexor surface of extremities of AD cases. AD patients were treated with topical corticosteroids (with or without antihistamines). Normal skin samples were obtained from nonlesional skin of healthy individuals who underwent skin surgery for benign skin lesions in the trunk and extremities. In all patients with AD and healthy individuals, there were no other past medical histories. Sections (5 μm) were deparaffinized and rehydrated through descending alcohol series and in phosphate buffered saline (PBS, pH 7.4). The sections were immersed in citrate buffer (pH 6.0) for 20 min at 95°C, and then immersed in 3% hydrogen peroxide in methanol to block endogenous peroxidase activity. The sections were immunohistochemically stained with anti-GCDFP15 mouse monoclonal antibody (1:25 dilution; Abcam, Cambridge, MA), anti-cholinergic receptor muscarin 3 rabbit polyclonal antibody (CHRM3; 1:100 dilution; Santa Cruz Biotechnology, Dallas, TX), and anti-acetylcholine esterase goat polyclonal (AchE; 1:50 dilution; Santa Cruz Biotechnology) antibody. The primary antibodies were incubated for 30 min at room temperature. After rinsing with PBS, sections were incubated for 30 min at room temperature with the secondary antibody, histofine simplestain MAX-PO (Nichirei Biosciences, Tokyo, Japan). The slides were then incubated with 3, 3-diamino-benzidine as the substrate for 3 min to visualize positively immunostained cells. Finally, the slides were counterstained with hematoxylin.

### Quantification of immunohistochemical staining

Digitalized section images were exported to JPEG files by NDP view software (Hamamatsu Photonics, Hamamatsu, Japan). The following processes were performed in Adobe Photoshop CS (Adobe Systems, San Jose, CA). Eccrine gland secretory coil cells were selected and expressed as Red channel histograms. Histograms revealed 255 different shades from pitch black (0) to pure white (255), and a number represented the level of brightness of each color. The staining intensities of 35 different areas were expressed as “red density” [23,24]. The areas were randomly selected from the eccrine gland secretory coil.

### Immunofluorescence staining

This study was in accordance with the Declaration of Helsinki. The sampling was approved by the Ethical Committee of Hamamatsu University School of Medicine, and written informed consent was obtained from all subjects. Skin samples were obtained by qualified dermatologists using a disposable sterile 3-mm punch biopsy (Maruho, Osaka, Japan) at the University Hospital of Hamamatsu University School of Medicine. AD skin samples were obtained from eczematous lesions in the trunk and flexor surface of extremities of AD cases. AD patients were treated by topical steroids (with or without antihistamines). Normal skin samples were obtained from nonlesional skin of healthy individuals who underwent skin surgery for benign skin lesions in the trunk and extremities. In all patients with AD and healthy individuals, there were no other past medical histories. Frozen skin specimens (6 μm thick) were incubated with each primary antibody at 4°C overnight. Anti-GCDFP15 mouse monoclonal antibody (1:100 dilution; Abcam), anti-carcinoembryonic antigen rabbit polyclonal antibody (CEA; 1:100 dilution; Abcam), anti-S100 rabbit polyclonal antibody (1:3 dilution; DAKO, Tokyo, Japan), and anti-CHRM3 rabbit polyclonal antibody (1:100 dilution; Santa Cruz Biotechnology) were diluted in PBS and used as primary antibody. The sections were then labeled for one hour with Alexa Fluor-488-conjugated donkey anti-mouse IgG (Molecular Probes, Eugene, OR) and Alexa Fluor-594-conjugated donkey anti-rabbit IgG (Molecular Probes, Eugene, OR) at 1:100 in PBS. Nuclei were stained with DAPI. Slides were washed and mounted on glycerol/PBS (1:1) for examination by fluorescence microscopy.

### Statistical analysis

The Mann-Whitney *U*-test was used to compare differences of GCDFP15 levels between AD and healthy individuals. *P* < 0.05 was considered significant. Two-way analysis of variance determined significance. The Student’s *t*-test was used to compare differences of red density between AD and healthy individuals.

## Results

### Quantification of GCDFP15 in SC and sweat samples

**Fig 1:**
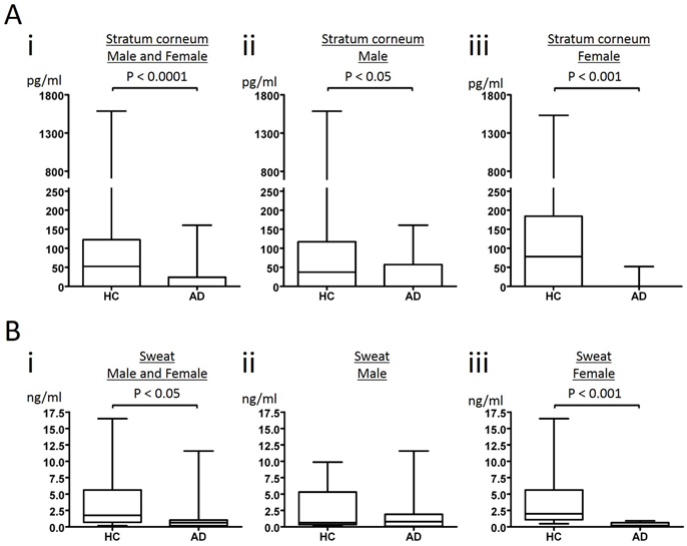
GCDFP15 levels in stratum corneum and sweat. Stratum corneum (SC) extracts (A) and sweat samples (B) were obtained from healthy control (HC) and atopic dermatitis (AD) individuals (i; male and female, ii; male, iii; female). GCDFP15 in SC extracts and sweat was quantified by ELISA. The results are expressed in box plot. The rectangle spans the first quartile to the third quartile. A segment inside the rectangle shows the median, and whiskers above and below the box show the locations of the minimum and maximum.

By proteome analysis using hybrid quadrupole-orbitrap mass spectrometer, we have shown that GCDFP15 is decreased in SC of AD patients [19]. Although proteome analysis can detect wide-ranging proteins, its procedure is too complicated to analyze a large number of samples. We therefore attempted to establish ELISA for quantification of GCDFP15 in SC extracts. SC samples were obtained by a non-invasive, tape-stripping method from 51 healthy control (HC) and 51 AD individuals, as previously reported [19]. The median values of HC and AD were 52.3 pg/ml and 0 pg/ml, respectively. The levels of GCDFP15 were significantly lower in AD than in HC (*p* < 0.0001, Fig 1A, i). Notably, the amounts of GCDFP15 were under detection level in 38 of 51 AD cases, while they were undetectable in 18 of 51 HC subjects. In male subjects, the median values of HC and AD were 37.4 pg/ml and 0 pg/ml, respectively (Fig 1A, ii). The levels of GCDFP15 were significantly lower in AD than in HC (*p* < 0.05). The amounts of GCDFP15 were under detection level in 21 of 31 AD cases, while they were undetectable in 11 of 29 HC subjects. In female subjects, the median values of HC and AD were 78.2 pg/ml and 0 pg/ml, respectively (Fig 1A, iii). The levels of GCDFP15 were significantly lower in AD than in HC (*p* < 0.001). The amounts of GCDFP15 were under detection level in 17 of 20 AD cases, while they were undetectable in 7 of 22 HC subjects.

We also analyzed GCDFP15 concentration in the sweat samples obtained from 18 HC and 12 AD individuals. The median values of HC and AD were 1.75 ng/ml and 0.63 ng/ml, respectively (Fig 1B, i). The GCDFP15 levels were undetectable in 2 men of 12 AD cases, while they could be measured in all HC samples. The GCDFP15 levels in sweat were significantly lower in AD than in HC (*p* < 0.05). In male subjects, the median values of HC and AD were 0.64 ng/ml and 0.80 ng/ml, respectively (Fig 1B, ii). We did not observe no significant differences in male subjects. In female subjects, the median values of HC and AD were 1.99 ng/ml and 0.39 ng/ml, respectively (Fig 1B, iii). The levels of GCDFP15 were significantly lower in AD than in HC (*p* < 0.001). The expression of GCDFP15 is regulated by the androgen receptor [14]. It is possible that the levels of androgen might affect the results of this analysis especially in male samples.

Since the median values of HC and AD were 52.3 pg/ml and 0 pg/ml in the SC samples, and 1.75 ng/ml and 0.63 ng/ml in the sweat samples, the difference between HC and AD was greater in the SC samples. Given that GCDFP15 secreted into sweat is accumulated in SC, these results suggest that the SC samples represent the accumulated amount of GCDFP15. It is known that eccrine sweating is attenuated in AD patients [7,8]. Taken together with the present results, it is considered that the low GCDFP15 amount in the SC of AD is attributable not only to low sweating but also to low concentration of GCDFP15 in sweat.

### Expression of GCDFP15 in normal and AD skin specimens

**Fig 2:**
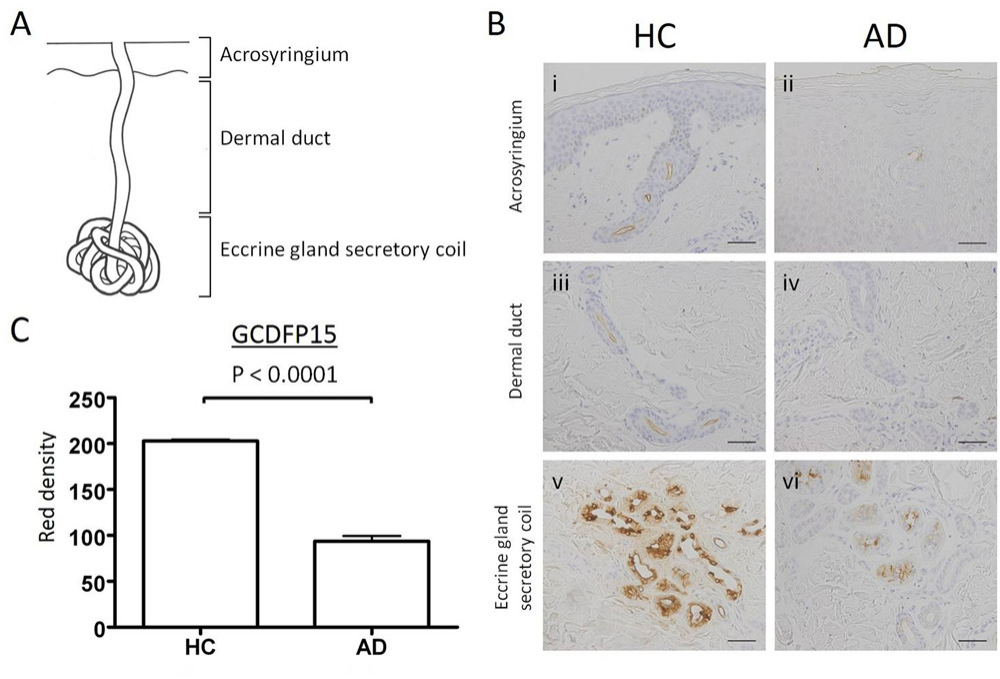
Immunohistochemical staining for GCDFP15 in eccrine sweat glands of normal and atopic dermatitis skin specimens. (A) Eccrine sweat glands are composed of eccrine gland secretory coil, dermal duct, and acrosyringium. (B) Normal and AD skin specimens were immunohistochemically stained with anti-GCDFP15 antibody. Bars indicate 50 μm. (C) The staining intensities are expressed as red density (RD). The RD of GCDFP15 in the eccrine gland secretory coil is significantly lower in AD patients. Results are expressed as mean ± SEM.

To identify the GCDFP15-producing cells in the skin, we performed an immunohistochemical study in normal and AD skin specimens. The eccrine sweat apparatus consists of eccrine gland secretory coil, dermal duct, and acrosyringium from the sweat-producing part to the sweat-secreting part (Fig 2A). In normal skin specimens, the eccrine gland secretory coil was strongly stained with anti-GCDFP15 antibody (Fig 2B, v). The positive staining was also observed in the lumen of dermal ducts (Fig 2B, iii) and acrosyringia (Fig 2B, i), and no epidermal keratinocytes were positively stained. In contrast, the expression levels of GCDFP15 were low in the eccrine gland secretory coil (Fig 2B, vi) and ducts (Fig 2B, ii and iv) of AD patients. To quantify the expression levels of GCDFP15, the staining intensity of GCDFP15 in 35 different areas of the eccrine gland secretory coil was monitored and expressed as “red density” (S1 Table) [23,24]. The mean ± SEM values of HC and AD were 202.9 ± 1.60 and 93.7 ± 5.75, respectively (Fig 2C). GCDFP15 expression was significantly decreased in AD skin compared to normal skin (*p* < 0.0001). These results confirmed low production of GCDFP15 in eccrine sweat glands of AD.

### GCDFP15-producing cells in eccrine glands

**Fig 3:**
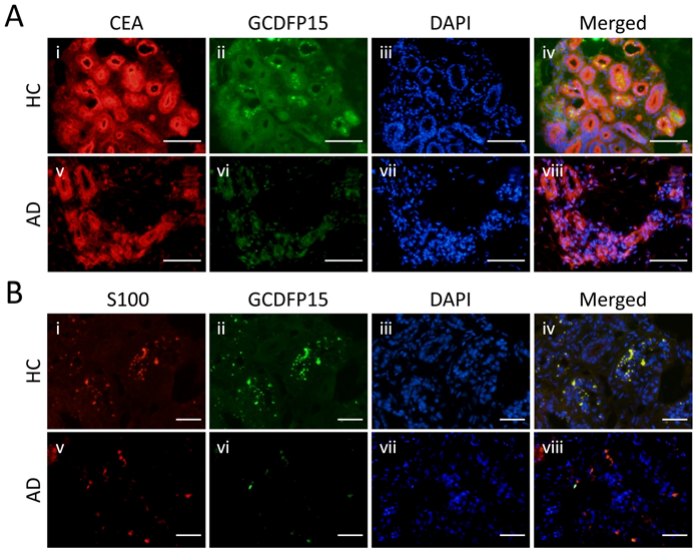
Distribution patterns of GCDFP15, CEA, and S100 protein in eccrine gland secretory coil. (A) Double staining of CEA and GCDFP15 in the eccrine gland secretory coil. The expression of CEA is observed in whole eccrine gland secretory coil cells. (B) Double staining of S100 protein and GCDFP15. The expression of S100 protein is observed in a part of eccrine gland secretory coil cells as granular pattern. Colocalization of S100 protein and GCDFP15 is shown in yellow in the merged image. Bars indicate 50 μm.

To identify the GCDFP15-producing cells, double immunofluorescence staining for sweat gland markers and GCDFP15 was performed in frozen specimens of normal and AD skin. First, CEA and GCDFP15 were double stained in the eccrine gland secretory coil (Fig 3A, i, v and ii, vi, respectively). Nuclei were stained with DAPI (Fig 3A, iii and vii). All eccrine gland secretory coil cells were positive for CEA, and the lumen of ductal cells was accentuated with CEA in both normal and AD specimens (Fig 3A, i and v). In contrast, positive GCDFP15 staining was observed as granular pattern in the eccrine gland secretory coil cells of normal skin (Fig 3A, ii). In AD lesional skin, however, the staining intensity of GCDFP15 was very low (Fig 3A, vi), suggesting poor production of GCDFP15.

Next, S100 protein and GCDFP15 were double stained in the eccrine gland secretory coil (Fig 3B, i, v and ii, vi, respectively). Nuclei were stained with DAPI (Fig 3B, iii and vii). There are two types of eccrine gland secretory coil cells, clear cell and dark cell [25]. The clear cell is positive for S100 protein, whereas the dark cell is S100 negative [26]. Similarly to GCDFP15 (Fig 3B, ii), S100 protein was positively stained as granular pattern in the eccrine gland secretory coil cells of normal skin (Fig 3B, i). The images of S100 and GCDFP15 were merged together and yielded yellow staining (Fig 3B, iv). In AD lesional skin, S100 protein was expressed by some of the eccrine gland secretory coil cells (Fig 3B, v), but again, GCDFP15 was poorly expressed (Fig 3B, vi). These results suggest that GCDFP15 is produced by the clear cell of eccrine sweat glands.

### Poor expression of CHRM3 in eccrine glands of AD

**Fig 4:**
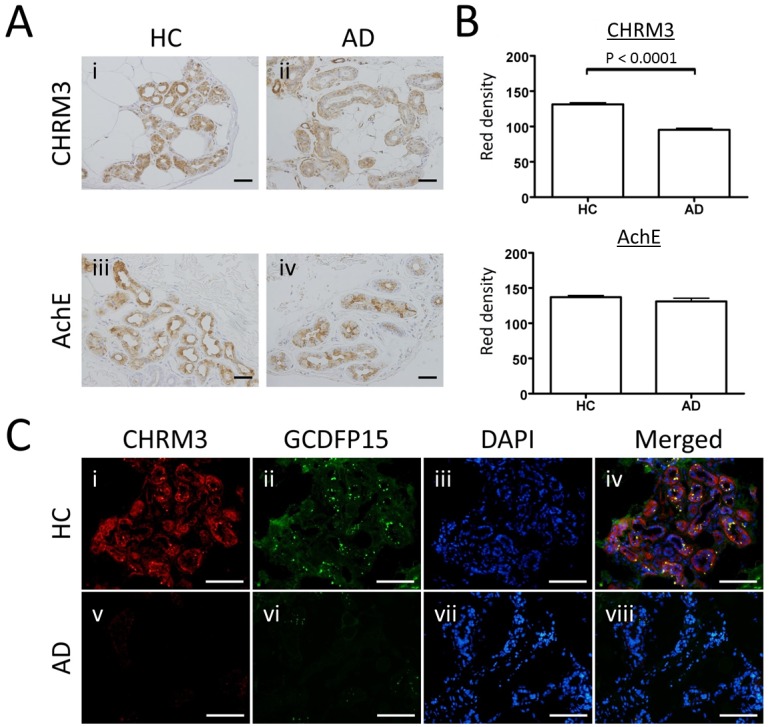
Expression of cholinergic receptor muscarin 3 and acetylcholine esterase in eccrine gland secretory coil of normal and atopic dermatitis skin specimens. (A) Representative immunohistochemical staining of cholinergic receptor muscarin 3 (CHRM3) and acetylcholine esterase (AchE). Bars indicate 50 μm. (B) The staining intensities are expressed as RD. The RD of CHRM3, but not AchE, is significantly lower in AD patients. Results are expressed as mean ± SEM. (C) Double immunofluorescence staining of CHRM3 and GCDFP15 in the eccrine gland secretory coil. In eccrine gland secretory coil cells, the expression levels of CHRM3 and GCDFP15 are decreased in parallel in AD. Bars indicate 50 μm.

Eccrine sweating is activated by acetylcholine, which binds to CHRM3 on eccrine gland secretory coil cells [3]. Excess acetylcholine is degraded by AchE produced by eccrine epithelial cells. Therefore, the expression degrees of CHRM3 and AchE may affect sweating. We have previously shown that hypohidrosis or anhidrosis associated with cholinergic urticaria is caused by attenuated expression of CHRM3 [23]. To address the mechanism underlying the decreased eccrine sweating in AD patients, we examined the expression of CHRM3 and AchE in the eccrine gland secretory coil of normal and AD skin specimens. Immunohistochemical staining showed that CHRM3 was expressed at low intensities in AD skin (Fig 4A, ii) compared to normal skin (Fig 4A, i). To quantify the expression levels of CHRM3, the staining intensity of CHRM3 in 35 different areas of the eccrine gland secretory coil was expressed as “red density” (S2 Table) [23,24]. The mean ± SEM values of HC and AD were 131.5 ± 2.31 and 95.3 ± 2.38, respectively (Fig 4B). CHRM3 expression was significantly decreased in AD skin compared to normal skin (*p* < 0.0001). In contrast, AchE expression was comparable between normal and AD specimens (Fig 4A, iii and iv). The mean ± SEM values of HC and AD were 137.1 ± 2.38 and 131.1 ± 4.39, respectively (Fig 4B, S3 Table). We did not observe no significant differences between HC and AD. Double staining for CHRM3 and GCDFP15 was also performed (Fig 4C, i, v and ii, vi, respectively). Nuclei were stained with DAPI (Fig 4C, iii and vii). Double staining for CHRM3 and GCDFP15 further confirmed that CHRM3 expression was attenuated in parallel with decreased GCDFP15 expression in AD patients (Fig 4C, viii). Thus, it is suggested that the attenuated eccrine sweating in AD is at least partly due to the decreased CHRM3 expression.

## Discussion

In this study, we established a method to quantify the GCDFP15 expression in SC by ELISA. In confirmation of our previous study [19], the amount of GCDFP15 was decreased in SC of AD patients as compared with HC subjects. We also found that the concentration of GCDFP15 was decreased in the patients’ sweat samples. Considering that AD patients usually have low levels of sweating [7,8], the reduced GCDFP15 amount in SC is caused by not only hypohidrosis but also attenuated GCDFP15 production. Thus, GCDFP15 is a potential monitoring marker for quantification and qualification of the sudomotor function. Since the difference in GCDFP15 amount between AD and HC was greater in SC than in sweat, the SC GCDFP15 value is thought to represent accumulation of GCDFP15 that is secreted from sweat and spreads to skin surface in a certain period. In this sense, GCDFP15 may be a relatively long-term marker to evaluate sweating.

Our double immunofluorescence study showed that the GCDFP15-producing cells were positive for S100 protein. Since S100 protein is expressed by the clear cell of the eccrine gland secretory coil [26], GCDFP15 seems to be secreted from the clear cell. Although the mechanism of GCDFP15 production by the clear cell remains unknown, it is possible that the protein production/secretion is associated with the sweating activity. The decreased amount of GCDFP15 in SC of AD is at least partly caused by the attenuated sweating. We, for the first time, demonstrated the low expression of CHRM3 in the eccrine glands of AD patients. The decreased expression of CHRM3 was also found in the anhidrotic skin of cholinergic uriticaria [23,27]. Considering that cholinergic urticaria is frequently associated with AD, these two disorders may share the hypohidrotic mechanism with each other.

It was reported that the direct sweat volume of lesional or non-lesional AD skin induced by direct stimulation with acetylcholine was only slightly reduced on the volar aspect of the forearm when compared with that in non-AD patients [11]. It is speculated that increased sweating might have been observed on the flexor aspect of the elbow or neck according to the compensation theory of sweating. In our present study, we observed the decreased expression of CHRM3 in the trunk and flexor surface of extremities of AD cases where eczematous lesions are commonly observed in AD. Therefore, we speculate that sweating by acetylcholine stimulation decreases in the trunk and flexor surface of extremities, then sweating elsewhere increases in compensation. This is one possible explanation about the past observation [11].

The function of GCDFP15 still remains unknown. It is reported that salivary GCDFP15 is associated with the aggregation of oral bacteria [28]. The GCDFP15-promoting bacterium aggregation may contribute to the oral defense system. Antimicrobial peptides, such as dermcidin, cathelicidin, and β-defensin, have a significant role in the skin defense. These peptides are selectively produced in sweat glands, secreted into the sweat and transported to the epidermal surface [29,30,31]. We hypothesized that GCDFP15 might be associated with the skin defense. We therefore attempted to evaluate the antimicrobial ability of recombinant GCDFP15 against the normal inhabitants of the skin. However, we did not find its antimicrobial activities against *Staphylococcus aureus*, *Streptococcus pyogenes*, and *Candida albicans* (data not shown). Further investigations are required to clarify the function of GCDFP15 in the skin.

Skin hydration is one of the central issues to elucidate the mechanism underlying barrier abnormalities and eczema development in AD. Recent studies have focused on the barrier impairment in AD and shown that allergic conditions may be preceded by skin barrier impairment or associated disorders of innate and acquired immunity [32]. Filaggrin gene mutation represents a typical cause of barrier impairment [33]. The barrier function is usually assessed by transepidermal water loss (TEWL) and skin surface hydration (capacitance), and AD patients have increased TEWL and lower skin surface hydration with dysregulation of proteinases and cytokines [34]. However, skin hydration by virtue of sweating is critical, as sweating is a major source of water in SC, and decreased sudomotor function may be involved in both the cause and aggravation of AD [7]. In this respect, evaluation of sweating is important for the AD state of individual patients.

In our previous [19] and present studies, SC samples were obtained by a non-invasive tape stripping technique. ELISA is more useful for quantification of GCDFP15 than proteome analysis. However, the method to extract protein from the SC-harvested tape is still complicated and comprised of multi-steps. Development of a more practical extraction procedure for ELISA may facilitate GCDFP15 to be a monitoring marker for eccrine sweating.

In conclusion, this study showed that the amount of GCDFP15 was decreased in the SC of AD patients. Accordingly, we found that the expression of GCDFP15 by eccrine gland secretory coil cells was reduced in AD lesional skin. GCDFP15 may be a possible marker of eccrine sweating for AD and other related diseases.

## Supporting Information

S1 Table Red density for GCDFP15. (DOC)

S2 Table Red density for cholinergic receptor muscarin 3. (DOC)

S3 Table Red density for acetylcholine esterase. (DOC)
