# Supplementary material for: Gross Cystic Disease Fluid Protein 15 in Stratum Corneum Is a Potential Marker of Decreased Eccrine Sweating for Atopic Dermatitis
Source: PLoS One. 2015 Apr 28;10(4):e0125082. doi: 10.1371/journal.pone.0125082 (PMC4412570; doi:10.1371/journal.pone.0125082)
Supplement: S1 Table — (DOC) [file pone.0125082.s001.doc]

**S1 Table. Red density for GCDFP15.**

| No | HC | AD | No | HC | AD | No | HC | AD |
| --- | --- | --- | --- | --- | --- | --- | --- | --- |
| 1 | 205 | 132 | 13 | 190 | 87 | 25 | 193 | 118 |
| 2 | 208 | 66 | 14 | 187 | 43 | 26 | 204 | 138 |
| 3 | 201 | 94 | 15 | 194 | 50 | 27 | 222 | 135 |
| 4 | 195 | 110 | 16 | 211 | 88 | 28 | 217 | 89 |
| 5 | 197 | 105 | 17 | 198 | 106 | 29 | 194 | 74 |
| 6 | 218 | 96 | 18 | 208 | 44 | 30 | 220 | 69 |
| 7 | 214 | 85 | 19 | 195 | 52 | 31 | 197 | 122 |
| 8 | 203 | 82 | 20 | 196 | 43 | 32 | 203 | 138 |
| 9 | 191 | 104 | 21 | 205 | 166 | 33 | 220 | 131 |
| 10 | 212 | 111 | 22 | 206 | 157 | 34 | 200 | 115 |
| 11 | 197 | 59 | 23 | 191 | 71 | 35 | 203 | 95 |
| 12 | 197 | 38 | 24 | 208 | 67 |  | | |

HC: healthy control

AD: atopic dermatitis
