# Supplementary material for: Gross Cystic Disease Fluid Protein 15 in Stratum Corneum Is a Potential Marker of Decreased Eccrine Sweating for Atopic Dermatitis
Source: PLoS One. 2015 Apr 28;10(4):e0125082. doi: 10.1371/journal.pone.0125082 (PMC4412570; doi:10.1371/journal.pone.0125082)
Supplement: S2 Table — (DOC) [file pone.0125082.s002.doc]

**S2 Table. Red density for cholinergic receptor muscarin 3.**

| No | HC | AD | No | HC | AD | No | HC | AD |
| --- | --- | --- | --- | --- | --- | --- | --- | --- |
| 1 | 141 | 126 | 13 | 138 | 129 | 25 | 136 | 125 |
| 2 | 147 | 106 | 14 | 143 | 95 | 26 | 120 | 93 |
| 3 | 147 | 86 | 15 | 137 | 68 | 27 | 141 | 92 |
| 4 | 131 | 91 | 16 | 115 | 116 | 28 | 160 | 80 |
| 5 | 101 | 81 | 17 | 128 | 86 | 29 | 123 | 82 |
| 6 | 151 | 87 | 18 | 121 | 94 | 30 | 129 | 82 |
| 7 | 131 | 96 | 19 | 111 | 108 | 31 | 124 | 89 |
| 8 | 148 | 112 | 20 | 143 | 95 | 32 | 110 | 99 |
| 9 | 113 | 112 | 21 | 128 | 84 | 33 | 131 | 90 |
| 10 | 146 | 90 | 22 | 131 | 85 | 34 | 149 | 104 |
| 11 | 122 | 92 | 23 | 112 | 100 | 35 | 137 | 84 |
| 12 | 130 | 91 | 24 | 127 | 84 |  | | |

HC: healthy control

AD: atopic dermatitis
