# Supplementary material for: Gross Cystic Disease Fluid Protein 15 in Stratum Corneum Is a Potential Marker of Decreased Eccrine Sweating for Atopic Dermatitis
Source: PLoS One. 2015 Apr 28;10(4):e0125082. doi: 10.1371/journal.pone.0125082 (PMC4412570; doi:10.1371/journal.pone.0125082)
Supplement: S3 Table — (DOC) [file pone.0125082.s003.doc]

**S3 Table. Red density for acetylcholine esterase.**

| No | HC | AD | No | HC | AD | No | HC | AD |
| --- | --- | --- | --- | --- | --- | --- | --- | --- |
| 1 | 147 | 103 | 13 | 140 | 119 | 25 | 124 | 131 |
| 2 | 135 | 143 | 14 | 121 | 150 | 26 | 126 | 155 |
| 3 | 127 | 156 | 15 | 155 | 155 | 27 | 122 | 86 |
| 4 | 137 | 121 | 16 | 157 | 111 | 28 | 157 | 100 |
| 5 | 133 | 117 | 17 | 133 | 116 | 29 | 154 | 160 |
| 6 | 134 | 150 | 18 | 115 | 143 | 30 | 130 | 98 |
| 7 | 171 | 104 | 19 | 120 | 93 | 31 | 121 | 84 |
| 8 | 153 | 162 | 20 | 160 | 156 | 32 | 123 | 134 |
| 9 | 141 | 161 | 21 | 143 | 102 | 33 | 135 | 115 |
| 10 | 131 | 121 | 22 | 138 | 157 | 34 | 119 | 154 |
| 11 | 141 | 162 | 23 | 134 | 150 | 35 | 157 | 148 |
| 12 | 122 | 171 | 24 | 143 | 102 |  | | |

HC: healthy control

AD: atopic dermatitis
